# Association of Maternal Comorbidity Burden With Cesarean Birth Rate Among Nulliparous, Term, Singleton, Vertex Pregnancies

**DOI:** 10.1001/jamanetworkopen.2023.38604

**Published:** 2023-10-19

**Authors:** Cara S. Wetcher, Rachel L. Kirshenbaum, Alejandro Alvarez, Rachel P. Gerber, Sarah L. Pachtman Shetty, Monique De Four Jones, Fernando Suarez, Adriann Combs, Michael Nimaroff, Dawnette Lewis, Matthew J. Blitz

**Affiliations:** 1 Department of Obstetrics and Gynecology, Donald and Barbara Zucker School of Medicine at Hofstra/Northwell, Hempstead, New York; 2 Biostatistics Unit, Office of Academic Affairs, Northwell Health, New Hyde Park, New York; 3 Katz Institute for Women’s Health, Northwell Health, New York, New York; 4 Institute of Health Systems Science, Feinstein Institutes for Medical Research, Northwell Health, Manhasset, New York

## Abstract

**IMPORTANCE:**

Cesarean birth rate among nulliparous, term, singleton, vertex (NTSV) pregnancies is a standard quality measure in obstetrical care. There are limited data on how the number and type of preexisting conditions affect mode of delivery among primigravidae, and it is also uncertain how maternal comorbidity burden differs across racial and ethnic groups and whether this helps to explain disparities in the NTSV cesarean birth rate.

**OBJECTIVE:**

To determine the association between obstetric comorbidity index (OB-CMI) score and cesarean delivery among NTSV pregnancies and to evaluate whether disparities in mode of delivery exist based on race and ethnicity group after adjusting for covariate factors.

**DESIGN, SETTING, AND PARTICIPANTS:**

This cross-sectional study of deliveries between January 2019 and December 2021 took place across 7 hospitals within a large academic health system in New York and included all NTSV pregnancies identified in the electronic medical record system. Exclusion criteria were fetal demise and contraindication to labor.

**EXPOSURE:**

The OB-CMI score. Covariate factors assessed included race and ethnicity group (American Indian or Alaska Native, Asian or Pacific Islander, Hispanic, non-Hispanic Black, non-Hispanic White, other or multiracial, and declined or unknown), public health insurance, and preferred language.

**MAIN OUTCOME AND MEASURES:**

Cesarean delivery.

**RESULTS:**

A total of 30 253 patients (mean [SD] age, 29.8 [5.4] years; 100% female) were included. Non-Hispanic White patients constituted the largest race and ethnicity group (43.7%), followed by Hispanic patients (16.2%), Asian or Pacific Islander patients (14.6%), and non-Hispanic Black patients (12.2%). The overall NTSV cesarean birth rate was 28.5% (n = 8632); the rate increased from 22.1% among patients with an OB-CMI score of 0 to greater than 55.0% when OB-CMI scores were 7 or higher. On multivariable mixed-effects logistic regression modeling, there was a statistically significant association between OB-CMI score group and cesarean delivery; each successive OB-CMI score group had an increased risk. Patients with an OB-CMI score of 4 or higher had more than 3 times greater odds of a cesarean birth (adjusted odds ratio, 3.14; 95% CI, 2.90-3.40) than those with an OB-CMI score of 0. Compared with non-Hispanic White patients, nearly all other race and ethnicity groups were at increased risk for cesarean delivery, and non-Hispanic Black patients were at highest risk (adjusted odds ratio, 1.43; 95% CI, 1.31-1.55).

**CONCLUSIONS AND RELEVANCE:**

In this cross-sectional study of patients with NTSV pregnancies, OB-CMI score was positively associated with cesarean birth. Racial and ethnic disparities in this metric were observed. Although differences in the prevalence of preexisting conditions were seen across groups, this did not fully explain variation in cesarean delivery rates, suggesting that unmeasured clinical or nonclinical factors may have influenced the outcome.

## Key Points

**Question:**

How is maternal comorbidity burden associated with cesarean delivery in nulliparous, term, singleton, vertex (NTSV) pregnancies, and do racial and ethnic group differences help to explain disparities in the cesarean birth rate?

**Findings:**

In this cross-sectional study of 30 253 patients with NTSV pregnancies, obstetric comorbidity index (OB-CMI) score was positively associated with cesarean delivery. Differences in OB-CMI scores were seen across racial and ethnic groups, but this did not fully explain variation in cesarean delivery rates.

**Meaning:**

In this study, the OB-CMI score was associated with NTSV cesarean birth rate and may provide further insight into the disparities that exist between racial and ethnic groups.

## Teaser

This cross-sectional study aims to determine the association between obstetric comorbidity index score and cesarean delivery among nulliparous, term, singleton, vertex pregnancies and to evaluate whether disparities in mode of delivery exist based on race and ethnicity group after adjusting for covariate factors.

## Introduction

Although cesarean delivery is a valuable and potentially lifesaving intervention in many cases, the rapid increase in use of the procedure in the early 2000s was without clear medical justification.^1^ Hospitals with higher cesarean delivery rates have higher rates of severe maternal morbidity (SMM).^2^ Maternal mortality occurs more frequently after cesarean birth than after vaginal birth.^3,4^ After cesarean birth, there is an increased risk of developing complications in future pregnancies, including placental abnormalities such as placenta previa and placenta accreta.^4^ Patients with nulliparous, term, singleton, vertex (NTSV) pregnancies are a critical target population for reducing the overall cesarean delivery rate.^5,6^ Reduction of cesarean birth in this group is considered a national priority, and it has become a standard quality measure in obstetrical care. Nulliparous, term, singleton, vertex pregnancies are thought to have the most favorable conditions for vaginal birth, and these patients have been described as “low risk,” a largely true but somewhat oversimplified characterization. Over recent decades, maternal age, obesity, and the frequency of other comorbidities have been rising among first-time mothers.^7,8,9^ Specifically, the prevalence of hypertension, diabetes, thyroid disorders, autoimmune disorders, asthma, and mental health conditions have substantially increased.^10,11^ Furthermore, certain neurological and cardiac conditions that were once considered rare or incompatible with pregnancy are now seen with increasing frequency in pregnant patients.^12^

It is well established that maternal comorbidity burden is associated with risk of SMM,^13^ but there are relatively limited data on how the number and type of preexisting conditions affect mode of delivery among first-time pregnant patients.^14,15^ The obstetric comorbidity index (OB-CMI) screening tool was originally developed and validated by Bateman et al^16^ to predict SMM using utilization claims data. This weighted scoring system was later revised by Easter et al^17^ to incorporate additional clinical data not reliably obtained in health care utilization data sets. The OB-CMI score has also been used to study other clinical outcomes, such as use of general anesthesia for cesarean delivery.^18^ Although many studies have examined the role of patient-level factors on NTSV cesarean delivery rates, based on our review of the literature and to our knowledge, no prior study has evaluated the association between this index and primary cesarean delivery, an outcome for which racial and ethnic disparities have been widely reported.^19,20,21,22,23^ Moreover, it remains unclear how the number of maternal comorbidities differ across race and ethnicity groups and whether this affects the rate of cesarean birth.

The primary objective of this study was to determine the association between maternal comorbidity burden, as quantified by OB-CMI score (calculated at time of admission), and cesarean delivery among NTSV pregnancies. There were 2 secondary objectives: (1) to evaluate whether disparities in mode of delivery exist based on race and ethnicity group after adjusting for covariate factors, and (2) to evaluate whether indication for cesarean delivery differed by OB-CMI and race and ethnicity groups. We hypothesized that there would be a positive association between OB-CMI score and cesarean delivery rate and that differences across race and ethnicity groups, if present, could be partially explained by differences in OB-CMI score.

## Methods

### Patient Population

This was a retrospective cross-sectional study of all patients with NTSV pregnancies who delivered between January 2019 and December 2021 at 7 hospitals within a large academic health system in New York. These facilities serve a diverse patient population that resides in both urban and suburban communities and represents the full socioeconomic spectrum. Inclusion criteria were NTSV pregnancies. Patients who did not meet the following NTSV criteria were excluded: multiparous patients, preterm deliveries (<37 weeks of gestational age), multiple gestations, and nonvertex presentations. Additional exclusion criteria were intrauterine fetal demise and contraindications to labor identified early in pregnancy, including placenta previa, vasa previa, prior myomectomy with disruption of endometrium, and suspected placenta accreta spectrum.

Clinical and demographic data were obtained from the inpatient electronic medical record system (Sunrise Clinical Manager [Allscripts]). Maternal comorbidities were identified by *International Statistical Classification of Diseases, Tenth Revision, Clinical Modification *codes and clinical documentation. Baseline demographic data included public health insurance (yes/no), preferred language English (yes/no), and self-identified race and ethnicity (American Indian or Alaska Native, Asian or Pacific Islander, Hispanic, non-Hispanic Black, non-Hispanic White, other or multiracial, and declined or unknown), which were selected from prespecified categories at the time of hospital admission. Clinical data included cesarean delivery (yes/no), indication for cesarean delivery, hospital, and limited perinatal outcomes.

The Northwell Health Institutional Review Board approved this study as minimal-risk research using data collected for routine clinical practice and thus waived the requirement for informed consent. The study followed the Strengthening the Reporting of Observational Studies in Epidemiology (STROBE) reporting guideline.

### Primary Outcome

The primary outcome was cesarean delivery, a binary variable. The OB-CMI score, the primary independent variable, was calculated at the time of admission for delivery hospitalization. This score is based on 24 weighted maternal comorbidity indicators as described by Easter et al.^17^ At Northwell Health, a proprietary web application accessible to clinical directors and operations managers was implemented and automatically calculates this metric. This robust tool does not rely exclusively on diagnosis codes but also incorporates clinical documentation, thereby reducing misclassification of comorbidities and outcomes. During the study period, OB-CMI scores were not displayed directly on the electronic medical record interface; therefore, clinicians were blinded to the OB-CMI scores of patients under their care. The OB-CMI scores ranged from 0 to 18 among the included patients with NTSV pregnancies, and more than half of the patients had a score of 0. Therefore, for statistical analysis, this variable was recoded as a categorical factor where patients with an OB-CMI score of 0 were one category, and the remaining patients were subdivided into quartiles. Nonetheless, due to the skewed distribution of OB-CMI scores, perfect quartiles were not achieved, and the variable denoting OB-CMI score was recoded into the following OB-CMI groups: 0, 1, 2, 3, and 4 or higher.

### Statistical Analysis

Descriptive statistics were used to characterize the demographic and clinical data. The χ^2^ test was used to examine associations between categorical variables. For analysis of the primary outcome, multivariable mixed effects logistic regression, which incorporates fixed and random effects, was used to model the probability of a cesarean delivery as a function of the OB-CMI score group, where observations were nested within the hospital in which they occurred. Logistic regression was first performed with the OB-CMI group as the only independent variable in the model (ie, the unadjusted model). An adjusted logistic regression model was then created by adding potential covariates: race and ethnicity group, public health insurance, and preferred language. Adjusted odds ratios (AORs) are presented along with the corresponding 95% CIs. Statistical significance was defined as 2-sided *P* < .05. The sample size for this study was based on availability of data from the inpatient database and not on any formal statistical power calculations. All analyses were conducted using SAS Enterprise Guide, release 3.8 (SAS Institute Inc). Because the study period was greatly affected by the COVID-19 pandemic, we evaluated whether there was an association between pandemic period (March 2020 onward) and cesarean birth.

## Results

**Figure 1.**
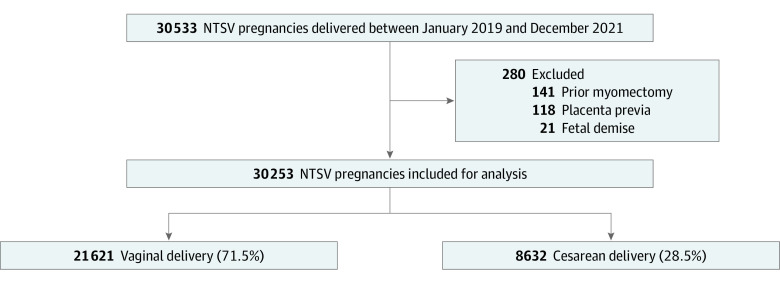
Cohort Flowchart NTSV indicates nulliparous, term, singleton, vertex.

A total of 30 253 patients (mean [SD] age, 29.8 [5.4] years; 100% female) with NTSV pregnancies were included for analysis (Figure 1). Non-Hispanic White patients constituted the largest race and ethnicity group (43.7%), followed by Hispanic patients (16.2%), Asian or Pacific Islander patients (14.6%), and non-Hispanic Black patients (12.2%). Baseline characteristics of the study population are summarized in Table 1 (stratified results are summarized in eTables 1 and 2 in Supplement 1). Most patients had private health insurance (72.8%), spoke English as their preferred language (94.4%), had no comorbidities (56.7%), and were younger than 35 years of age at delivery (81.2%). The most common obstetric comorbidities were maternal age of 35 to 39 years (15.6%), preeclampsia/gestational hypertension/chronic hypertension (12.8%), asthma (6.7%), body mass index (BMI; calculated as weight in kilograms divided by height in meters squared) of 40 to 49.9 (6.0%), and preeclampsia with severe features or eclampsia (5.1%). The overall NTSV cesarean delivery rate was 28.5% (n = 8632); this rate varied from 18.4% to 32.5% across the 7 included hospitals (*P* < .001). Cesarean births increased after the onset of the COVID-19 pandemic, from 27.4% to 29.2% (*P* < .001).

**Table 1.** Baseline Demographics and Comorbidities (N = 30 253)

Characteristic	No. (%)
Race and ethnicity^a^	
American Indian or Alaska Native	188 (0.6)
Asian or Pacific Islander	4422 (14.6)
Hispanic	4896 (16.2)
Non-Hispanic Black	3683 (12.2)
Non-Hispanic White	13 217 (43.7)
Other or multiracial	2782 (9.2)
Declined or unknown	1065 (3.5)
Has public health insurance	8227 (27.2)
Preferred language is English	28 569 (94.4)
Comorbidity (points in OB-CMI score)^b^	
Preeclampsia with severe features or eclampsia (5)	1550 (5.1)
Preeclampsia/gestational/chronic hypertension (2)	3877 (12.8)
Congestive heart failure (5)	12 (<0.1)
Pulmonary hypertension (4)	13 (<0.1)
Ischemic heart disease/cardiac arrhythmia (3)	89 (0.3)
Congenital heart and/or valvular disease (4)	93 (0.3)
Placental abruption (4)	164 (0.5)
Autoimmune disease/lupus (2)	521 (1.7)
HIV/AIDS (2)	19 (0.1)
Sickle cell disease/bleeding disorder/coagulopathy/anticoagulation (3)	707 (2.3)
Epilepsy/cerebrovascular accident/neuromuscular disorder (2)	221 (0.7)
Chronic kidney disease (1)	12 (<0.1)
Asthma (1)	2030 (6.7)
Diabetes requiring insulin (1)	404 (1.3)
Maternal age, y	
>44 (3)	115 (0.4)
40-44 (2)	847 (2.8)
35-39 (1)	4724 (15.6)
Substance use disorder (2)	273 (0.9)
Alcohol abuse (1)	90 (0.3)
BMI	
>50 (3)	332 (1.1)
40-49.9 (2)	1809 (6.0)

Abbreviations: BMI, body mass index (calculated as weight in kilograms divided by height in meters squared); OB-CMI, obstetric comorbidity index.

^a^ Race and ethnicity were self-identified from prespecified categories at the time of hospital admission.

^b^ OB-CMI components excluded from study include multiple gestation, intrauterine fetal demise, placenta previa/suspected accreta, and previous cesarean delivery/myomectomy.

**Figure 2.**
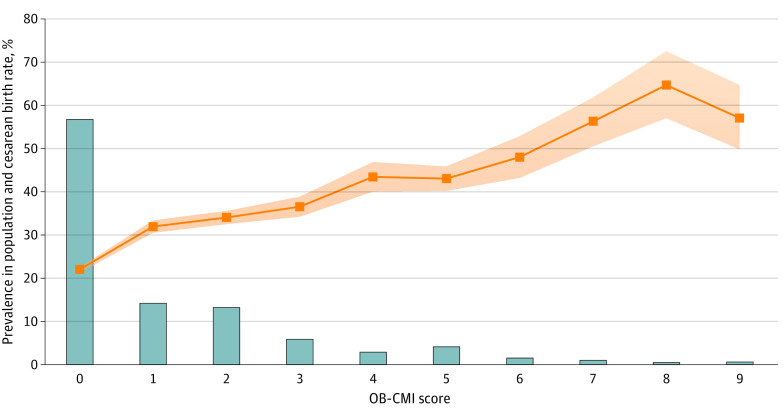
Distribution of OB-CMI Scores and Corresponding Cesarean Birth Rates Among Nulliparous, Term, Singleton, Vertex Pregnancies Bars represent the prevalence of each obstetric comorbidity index (OB-CMI) score in the cohort, and data points represent the cesarean birth rate, with shaded area indicating 95% CIs.

Figure 2 shows the prevalence of each OB-CMI score within the cohort and corresponding NTSV cesarean delivery rates. More than half of patients (56.7%) had no comorbidities that are components of the OB-CMI score. The cesarean birth rate increased from 22.1% among patients with an OB-CMI score of 0 to greater than 55% when OB-CMI scores were 7 or higher.

On unadjusted analysis, there was a statistically significant association between OB-CMI score group and cesarean delivery. Results of multivariable mixed-effects logistic regression modeling are summarized in Table 2. Each successive OB-CMI group had an increased risk, and patients with an OB-CMI score of 4 or higher had more than 3 times greater odds of a cesarean birth compared with patients with an OB-CMI score of 0 (AOR, 3.14; 95% CI, 2.90-3.40). The AORs for OB-CMI score groups did not change appreciably from the crude ORs in the unadjusted model. Compared with non-Hispanic White patients, all other race and ethnicity groups except for American Indian or Alaska Native (n = 188) were at increased risk for cesarean delivery after adjusting for other explanatory variables; non-Hispanic Black patients were at highest risk (AOR, 1.43; 95% CI, 1.31-1.55). Patients with public health insurance had a 19.4% lower odds of cesarean delivery (AOR, 0.81; 95% CI, 0.75-0.86) compared with those without public health insurance. Preferred language of English was associated with 17.6% higher odds of cesarean delivery (AOR, 1.18; 95% CI, 1.03-1.34). When the COVID-19 pandemic period (yes/no) was included as a covariate in the regression model (AOR, 1.06; 95% CI, 1.00-1.12), the other results were nearly identical with each AOR, differing by 0.01. Given the very small effect size, pandemic period was not included in the final model.

**Table 2.** Multivariable Mixed-Effects Logistic Regression Model for Cesarean Delivery Among NTSV Pregnancies After Adjusting for Covariate Factors

Covariate factor	Delivery, No. (%)		Odds ratio (95% CI)	Adjusted odds ratio (95% CI)
	Cesarean (n = 8632)	Vaginal (n = 21 621)		
OB-CMI score				
0	3762 (22.1)	13 293 (77.9)	1 [Reference]	1 [Reference]
1	1368 (32.0)	2908 (68.0)	1.62 (1.51-1.75)	1.58 (1.47-1.70)
2	1356 (34.1)	2615 (65.9)	1.83 (1.70-2.98)	1.80 (1.66-1.94)
3	644 (36.6)	1115 (63.4)	2.04 (1.84-2.27)	1.99 (1.79-2.21)
≥4	1502 (47.1)	1690 (52.9)	3.23 (2.99-3.50)	3.14 (2.90-3.40)
Race and ethnicity^a^				
American Indian or Alaska Native	50 (26.6)	138 (73.4)	0.98 (0.70-1.35)	1.33 (0.95-1.86)
Asian or Pacific Islander	1194 (27.0)	3228 (73.0)	1.00 (0.95-1.06)	1.12 (1.03-1.21)
Hispanic	1371 (28.0)	3525 (72.0)	1.05 (0.98-1.13)	1.19 (1.10-1.30)
Non-Hispanic Black	1273 (34.6)	2410 (65.4)	1.43 (1.32-1.55)	1.43 (1.31-1.55)
Non-Hispanic White	3566 (27.0)	9651 (73.0)	1 [Reference]	1 [Reference]
Other or multiracial	848 (30.5)	1934 (69.5)	1.19 (1.09-1.30)	1.29 (1.17-1.42)
Declined or unknown	330 (31.0)	735 (69.0)	1.22 (1.06-1.39)	1.18 (1.02-1.35)
Health insurance				
Not public	6600 (30.0)	15 426 (70.0)	1 [Reference]	1 [Reference]
Public	2032 (24.7)	6195 (75.3)	0.77 (0.72-0.81)	0.81 (0.75-0.86)
Preferred language				
Not English	396 (23.5)	1288 (76.5)	1 [Reference]	1 [Reference]
English	8236 (28.8)	20 333 (71.2)	1.32 (1.17-1.48)	1.18 (1.03-1.34)

Abbreviations: NTSV, nulliparous, term, singleton, vertex; OB-CMI, obstetric comorbidity index.

^a^ Race and ethnicity were self-identified from prespecified categories at the time of hospital admission.

**Figure 3.**
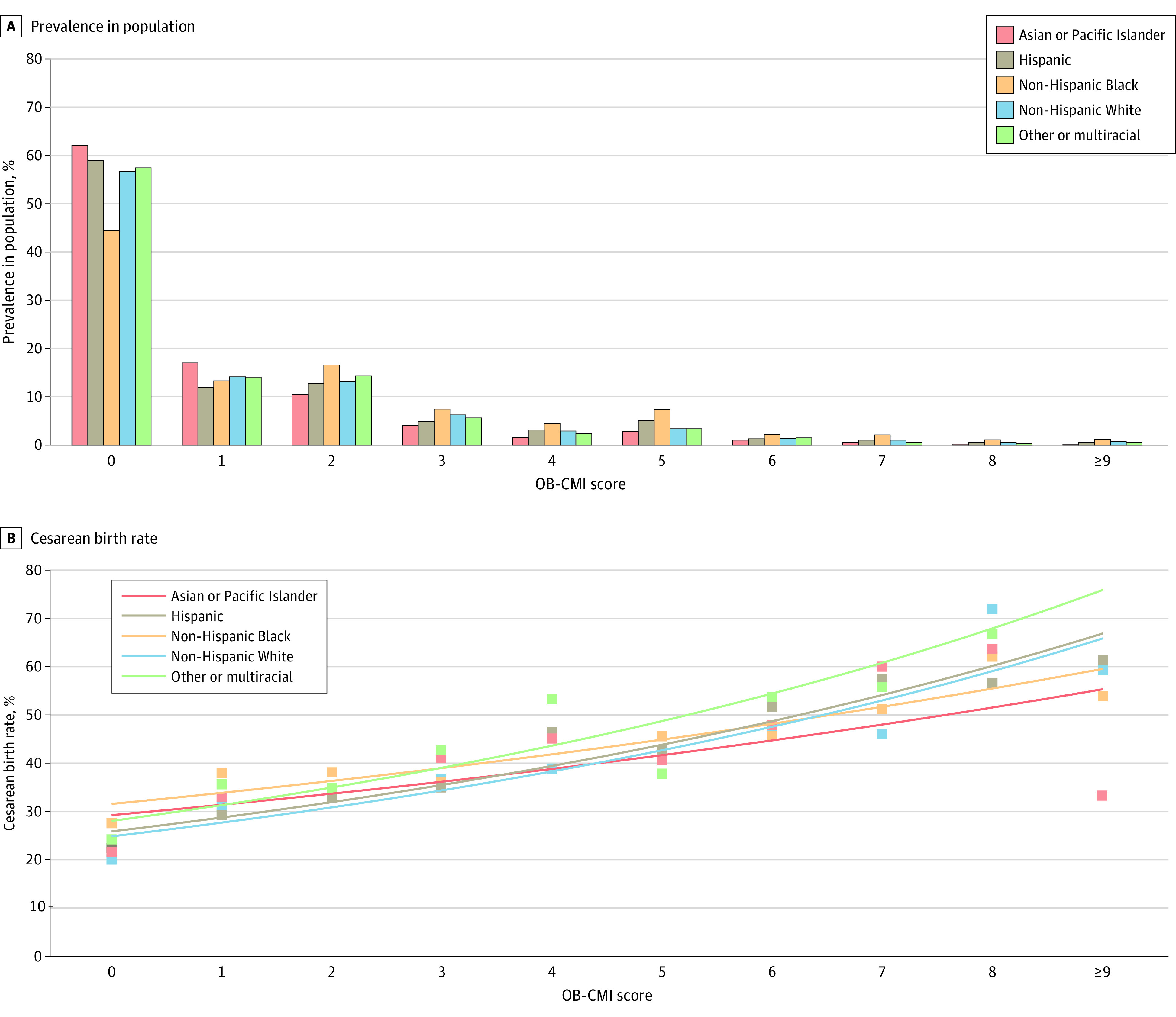
Cesarean Birth Rate by Race and Ethnicity Group and OB-CMI Score Among Nulliparous, Term, Singleton, Vertex Pregnancies Race and ethnicity were self-identified from prespecified categories at the time of hospital admission. The American Indian or Alaska Native group is not shown due to low sample size. Bars represent the prevalence of each obstetric comorbidity index (OB-CMI) score in the cohort (A), and data points represent the cesarean birth rate, with best-fit trendlines shown to facilitate comparisons between groups (B).

Figure 3 shows NTSV cesarean delivery rates for each race and ethnicity group stratified by OB-CMI score. A majority of non-Hispanic Black patients had 1 or more comorbidities (55.6%). For all other race and ethnicity groups, fewer than half of the patients had comorbidities. Compared with non-Hispanic White patients, non-Hispanic Black patients were 1.6 times more likely to have 1 or more comorbidities (OR, 1.64; 95% CI, 1.53-1.77).

Among low-risk pregnancies without any maternal comorbidities (OB-CMI score of 0), cesarean birth rates differed when comparing race and ethnicity groups. Within this healthier subgroup (n = 17 055), non-Hispanic Black patients had the highest rate of cesarean birth (450 of 1634 [27.5%]). The lowest cesarean delivery rates were seen among non-Hispanic White patients (1516 of 7497 [21.7%]) and American Indian or Alaska Native patients (22 of 117 [18.8%]). Interestingly, among higher OB-CMI groups (score of ≥3), there were no statistically significant differences in cesarean delivery rates when comparing race and ethnicity groups.

The most common indication for cesarean birth was abnormal fetal status (4328 [50.2%]; eTable 3 in Supplement 1), which was seen more often for patients with an OB-CMI score of 0 compared with those with an OB-CMI score of 4 or higher (53.7% vs 43.0%, respectively; *P* < .001). Non-Hispanic Black patients had statistically significant higher rates of abnormal fetal status than non-Hispanic White patients both overall (61.2% vs 44.4%, respectively) and across all OB-CMI score groups (eTable 4 in Supplement 1).

Limited perinatal outcomes were evaluated. Low birth weight, small-for-gestational-age newborns, 5-minute Apgar score less than 7, and postpartum hemorrhage requiring blood transfusion all occurred more frequently for patients in higher OB-CMI score groups (eTable 5 in Supplement 1).

## Discussion

### Principal Findings

We observed that OB-CMI score was positively associated with cesarean delivery in a large, diverse population of patients with NTSV pregnancies. This may be characterized as a dose-response association between maternal comorbidity burden and cesarean delivery. Statistically significant differences in NTSV cesarean delivery rates were seen across race and ethnicity groups even after adjustment for preexisting maternal conditions. Specifically, among low-risk NTSV pregnancies without any maternal comorbidities (OB-CMI score of 0), non-Hispanic Black patients were at highest risk for cesarean birth. Interestingly, among higher OB-CMI groups (score of ≥3), cesarean birth rates did not differ when comparing race and ethnicity groups. Overall, non-Hispanic Black patients had more comorbidities than patients from other race and ethnicity groups and were more likely to have cesarean deliveries performed for abnormal fetal status regardless of OB-CMI score.

### Results in the Context of What Is Known

Previous studies have evaluated the association of maternal comorbidities with NTSV cesarean birth rates,^14,20,24^ and some have also examined scoring systems that summarize cumulative comorbidity burden.^15^ In contrast, the present approach builds on and enhances prior efforts by using a robust, validated comorbidity index that weighs diagnoses that reflect risk for SMM. In a national sample of NTSV pregnancies from 2016 to 2018, Andrikopoulou et al^14^ observed an association between BMI, maternal age, and clinical factors such as diabetes and hypertension with cesarean birth. Other investigators have reported similar findings and acknowledged the additive effect of such characteristics.^15^ One challenge in comparing such studies is that clinical groupings and definitions are inconsistent. Some authors classify overweight/obesity (BMI ≥25) as a comorbidity, whereas OB-CMI only incorporates class 3 obesity (BMI ≥40) and further differentiates the higher-risk subgroup with BMI greater than 50. Similarly, some authors classify any form of diabetes in pregnancy as a comorbidity, whereas OB-CMI only includes diabetes requiring insulin. More restrictive definitions may allow better identification of high-risk patients.

The present finding that non-Hispanic White patients with NTSV pregnancies had a lower cesarean delivery rate compared with most other race and ethnicity groups is consistent with the findings reported by Okwandu and colleagues^20^ in Northern California from 2016 to 2017, Edmonds and colleagues^25^ in Massachusetts from 2006 to 2011, and Min and colleagues^26^ in Maryland from 2004 to 2010. Despite different populations, geographies, clinical settings, and practice patterns, this finding persists. Furthermore, several investigators have reported that indications for cesarean delivery differ by racial and ethnic group, just as we report herein. Compared with non-Hispanic White patients, those belonging to other race and ethnicity groups have a greater odds of cesarean delivery for nonreassuring fetal status.^21,27^ Based on the present findings, the frequency of cesarean birth for abnormal fetal status varies not only by race and ethnicity group, but also by OB-CMI score, overall occurring less often when OB-CMI score is 4 or higher.

### Clinical Implications

The national NTSV cesarean delivery rate reported by the US Centers for Disease Control and Prevention increased to 26.3% in 2021.^28^ Healthy People 2030, the 10-year plan published by the US Department of Health and Human Services for addressing the country’s most critical public health challenges, recommends a target rate of 23.6%.^29^ Unfortunately, there is no evidence-based optimal cesarean delivery rate shown to minimize adverse maternal and neonatal outcomes in all populations.^30^ Complex risk adjustments would be required to compare rates across institutions.^31^ Target rates established by health care quality organizations are often arbitrary and do not consider differences in patient characteristics. Based on the present findings, we expect that the cesarean delivery rate would be higher in a population with more comorbidities, regardless of whether those cesarean deliveries are necessarily indicated. The goal for such a population should be simultaneous efforts to reduce comorbidities and cesarean births. Establishing and incentivizing unattainable quality measures could have unexpected negative effects.

Several strategies have been proposed to safely lower the rate of primary cesarean delivery.^4^ Interventions have been directed at patients (educational programs, childbirth workshops), health care professionals (peer review, mandatory second opinion, audit and feedback, review of evidence-based guidelines), and facilities (different staffing models), with varying success.^32^ There is evidence that standardized, evidence-based, induction and labor management protocols reduce the rate of cesarean delivery, particularly in racial and ethnic minority groups.^33^ Further efforts should be made to reduce modifiable risk factors and optimize chronic conditions before conception or early in gestation. In addition, nonclinical factors that may affect labor management must be explored, including social determinants of health, implicit bias, and structural as well as institutional racism.

### Research Implications

Given that racial and ethnic disparities in the NTSV cesarean delivery rate cannot be fully explained by differences in the clinical characteristics evaluated herein, future studies should seek to understand whether these results are due to other unmeasured clinical or nonclinical factors. In terms of nonclinical factors, provider-level, hospital-level, and system-level characteristics should be considered.

Physician characteristics have been shown to affect cesarean birth rates.^34^ The obstetrician’s training and experience, adherence to evidence-based guidelines, and personal beliefs and attitudes can all affect clinical decision-making. Implicit bias may also affect decisions regarding labor management and mode of delivery; this is a process whereby a person’s unconscious beliefs and attitudes affect their decisions and actions, often without their knowledge. Unconscious bias training may help reduce the likelihood that such bias will affect management decisions. Hospital and health system policies and resources may affect birth outcomes. Specifically, access to specialists, wait times, and use of health information technology all affect patient care.

Investigators should seek to understand whether first-time pregnant patients living in more socially vulnerable communities are at increased risk for cesarean birth. Pregnancy outcomes may be affected by reduced access to health care services (eg, contributing to insufficient comorbidity care), socioeconomic stressors (eg, poverty, crime, social isolation), and environmental factors (eg, pollution). Geographic localization of at-risk areas may allow for targeted public health interventions.

These findings should prompt further investigation into how to reduce modifiable risk factors in pregnant patients, particularly obesity, which itself is associated with numerous other comorbidities. Finally, the effect of longitudinal, multidisciplinary care coordination and delivery planning on racial and ethnic disparities in cesarean birth rates should be studied.

### Limitations

This study is limited by its retrospective nature and use of administrative data. Manual medical record review was not performed to determine the accuracy of automated comorbidity identification for this study, but the database from which the diagnoses were retrieved is routinely used for research and quality-improvement purposes. The OB-CMI was not developed to predict cesarean delivery; the factors included in this index and the relative weights assigned to those factors may differ from those associated with cesarean delivery risk. A more specific composite index may have stronger association with the primary outcome. In addition, fetal factors that may affect mode of delivery, such as growth restriction, were not evaluated. Restriction of data collection to a single health system may limit the generalizability of the present results to other geographic regions with different patient populations and clinical practice patterns. We did not evaluate the quality or consistency of prenatal care or how any chronic conditions were managed during pregnancy. It is not known if patients who received consistent subspecialty care or comanagement of conditions by maternal-fetal medicine had a reduced cesarean birth rate. We did not evaluate whether any characteristics of individual health care team members were associated with mode of delivery. Institutional characteristics were similarly not evaluated. Although we excluded patients with contraindications to labor, we did not evaluate whether any of the included patients were not offered a trial of labor. Finally, we did not analyze subgroups of patients based on labor-management decisions, nor did we classify patients with the 10-Group Classification System,^35,36^ in part because these intrapartum factors (induction or augmentation vs spontaneous labor) are mediators between exposure (OB-CMI) and outcome (cesarean birth). Mediators should not be adjusted for when examining the total effect of an exposure on an outcome; overadjustment bias occurs when controlling for intermediate variables.^37,38,39^

## Conclusions

In this retrospective cross-sectional study performed within a large academic health system in New York, maternal comorbidity burden, as quantified by OB-CMI score, was associated with an increased risk of NTSV cesarean birth. Racial and ethnic disparities in this metric exist. Although differences in the prevalence of preexisting conditions were seen across groups, this did not fully explain variation in the cesarean delivery rate, suggesting that unmeasured clinical or nonclinical factors are influencing the outcome. The contribution of structural racism, implicit bias, and social determinants of health must be considered when evaluating this quality metric. For most pregnant patients with comorbidities at term, vaginal delivery is the safest option. Therefore, we must better understand why the vaginal delivery rate is so low among the highest-risk patients and what we can do to prevent primary cesarean births among individuals for whom a vaginal delivery is not contraindicated.

## Notes

Supplement 1. **eTable 1.** Baseline demographics and comorbidities by obstetrical comorbidity index (OB-CMI) score group **eTable 2.** Baseline demographics and comorbidities by race and ethnicity group **eTable 3.** Cesarean delivery indications stratified by obstetrical comorbidity index (OB-CMI) score group and race and ethnicity group **eTable 4.** Cesarean delivery for abnormal fetal status stratified by obstetrical comorbidity index (OB-CMI) score group and race and ethnicity group **eTable 5.** Perinatal outcomes by obstetrical comorbidity index (OB-CMI) score group

Supplement 2.
Data Sharing Statement
