# Supplementary material for: Association of Maternal Comorbidity Burden With Cesarean Birth Rate Among Nulliparous, Term, Singleton, Vertex Pregnancies
Source: JAMA Netw Open. 2023 Oct 19;6(10):e2338604. doi: 10.1001/jamanetworkopen.2023.38604 (PMC10587795; doi:10.1001/jamanetworkopen.2023.38604)
Supplement: Supplement 1. — eTable 1. Baseline demographics and comorbidities by obstetrical comorbidity index (OB-CMI) score group eTable 2. Baseline demographics and comorbidities by race and ethnicity group eTable 3. Cesarean delivery indications stratified by obstetrical comorbidity index (OB-CMI) score group and race and ethnicity group eTable 4. Cesarean delivery for abnormal fetal status stratified by obstetrical comorbidity index (OB-CMI) score group and race and ethnicity group eTable 5. Perinatal outcomes by obstetrical comorbidity index (OB-CMI) score group [file jamanetwopen-e2338604-s001.pdf]

Supplemental Online Content

Wetcher CS, Kirshenbaum RL, Alvarez A, et al. Association of maternal comorbidity burden with cesarean birth rate among nulliparous, term, singleton, vertex pregnancies. *JAMA Netw Open*. 2023;6(10):e2338604. doi:10.1001/jamanetworkopen.2023.38604

eTable 1. Baseline demographics and comorbidities by obstetrical comorbidity index (OB-CMI) score group
eTable 2. Baseline demographics and comorbidities by race and ethnicity group
eTable 3. Cesarean delivery indications stratified by obstetrical comorbidity index (OB-CMI) score group and race and ethnicity group
eTable 4. Cesarean delivery for abnormal fetal status stratified by obstetrical comorbidity index (OB-CMI) score group and race and ethnicity group
eTable 5. Perinatal outcomes by obstetrical comorbidity index (OB-CMI) score group

This supplemental material has been provided by the authors to give readers additional information about their work.

© 2023 Wetcher CS et al. *JAMA Netw Open.*

eTable 1. Baseline demographics and comorbidities by obstetrical comorbidity index (OB-CMI) score group

Characteristic	OB-CMI Score Group				
	0 (n=17,055)	1 (n=4,276)	2 (n=3,971)	3 (n=1,759)	≥ 4 (n=3,192)
Race and ethnicity					
American Indian or Alaskan Native	117 (0.7)	28 (0.65)	26 (0.7)	5 (0.3)	12 (0.4)
Asian or Pacific Islander	2,748 (16.1)	753 (17.6)	462 (11.6)	179 (10.2)	280 (8.8)
Declined or unknown	578 (3.4)	168 (3.9)	122 (3.1)	81 (4.6)	116 (3.6)
Hispanic	2,883 (16.9)	582 (13.6)	625 (15.7)	242 (13.8)	564 (17.7)
Non-Hispanic Black	1,634 (9.6)	491 (11.5)	610 (15.4)	277 (15.7)	671 (21.0)
Non-Hispanic White	7,497 (43.9)	1,862 (43.5)	1,729 (43.5)	820 (46.6)	1,309 (41.0)
Other or multiracial	1,598 (9.4)	392 (9.2)	391 (9.8)	155 (8.8)	240 (7.5)
Public health insurance	5,113 (29.9)	745 (17.4)	1,058 (26.6)	398 (22.6)	913 (28.6)
Preferred language English	15,849 (92.9)	4,145 (96.9)	3,802 (95.7)	1,710 (97.2)	3,063 (95.9)
Comorbidity (points in OB-CMI score)[a]					
Preeclampsia with severe features or eclampsia (5)	-	0 (0.0)	0 (0.0)	0 (0.0)	1,550 (48.6)
Preeclampsia/gestational/chronic hypertension (2)	-	0 (0.0)	2,111 (53.2)	773 (43.9)	993 (31.1)
Congestive heart failure (5)	-	0 (0.0)	0 (0.0)	0 (0.0)	12 (0.4)
Pulmonary hypertension (4)	-	0 (0.0)	0 (0.0)	0 (0.0)	13 (0.4)
Ischemic heart disease/ cardiac arrhythmia (3)	-	0 (0.0)	0 (0.0)	33 (1.9)	56 (1.8)
Congenital heart and/or valvular disease (4)	-	0 (0.0)	0 (0.0)	0 (0.0)	93 (2.9)
Placental abruption (4)	-	0 (0.0)	0 (0.0)	0 (0.0)	164 (5.1)
Autoimmune disease/lupus (2)	-	0 (0.0)	185 (4.7)	75 (4.3)	261 (8.2)
HIV/AIDS (2)	-	0 (0.0)	7 (0.18)	3 (0.17)	9 (0.3)
Sickle cell disease/bleeding disorder/coagulopathy/anticoagulation (3)	-	0 (0.0)	0 (0.0)	308 (17.5)	399 (12.5)
Epilepsy/cerebrovascular accident/neuromuscular disorder (2)	-	0 (0.0)	68 (1.7)	17 (1.0)	136 (4.3)
Chronic renal disease (1)	-	1 (0.02)	1 (0.03)	2 (0.1)	8 (0.3)
Asthma (1)	-	1,018 (23.8)	172 (4.3)	385 (21.9)	455 (14.3)

© 2023 Wetcher CS et al. *JAMA Netw Open.*

Diabetes on insulin (1)	-	148 (3.5)	56 (1.4)	66 (3.8)	134 (4.2)
Maternal age, years	-				
> 44 (3)	-	0 (0.0)	0 (0.0)	59 (3.4)	56 (1.7)
40-44 (2)	-	0 (0.0)	533 (13.4)	43 (2.4)	271 (8.5)
35-39 (1)	-	3,081 (72.1)	205 (5.2)	746 (42.4)	692 (21.2)
Substance use disorder (2)	-	0 (0.0)	100 (2.5)	50 (2.8)	123 (3.9)
Alcohol abuse (1)	-	28 (0.65)	12 (0.3)	21 (1.2)	29 (0.9)
Body mass index (BMI), kg/m^2	-				
>50 (3)	-	0 (0.0)	0 (0.0)	143 (8.2)	189 (5.9)
40-49.9 (2)	-	0 (0.0)	743 (18.7)	253 (14.4)	813 (25.5)

Data are presented as n (%)
AIDS, acquired immunodeficiency syndrome; HIV, human immunodeficiency virus; OB-CMI, obstetric comorbidity index
[a] OB-CMI components excluded from study: multiple gestation, intrauterine fetal demise, placenta previa/suspected accreta, previous cesarean delivery/myomectomy

© 2023 Wetcher CS et al. *JAMA Netw Open.*

eTable 2. Baseline demographics and comorbidities by race and ethnicity group

**Characteristic**	**American Indian or Alaskan Native** (n=188)	**Asian or Pacific Islander** (n=4,422)	**Hispanic** (n=4,896)	**Non-Hispanic Black** (n=3,683)	**Non-Hispanic White** (n=13,217)	**Other or Multiracial** (n=2,782)	**Declined or Unknown** (n=1,065)
Public health insurance	93 (49.4)	1,120 (25.3)	2,568 (54.5)	1,219 (33.1)	1,999 (15.1)	932 (33.5)	296 (27.8)
Preferred language English	177 (94.1)	4,219 (95.4)	3,794 (77.5)	3,666 (99.5)	13,066 (98.9)	2,638 (94.8)	1,009 (94.7)
Comorbidity (points in OB-CMI score)[a]							
Preeclampsia with severe features or eclampsia (5)	10 (5.3)	160 (3.6)	299 (6.1)	342 (9.3)	567 (4.3)	119 (4.3)	53 (5.0)
Preeclampsia/gestational/chronic hypertension (2)	16 (8.5)	407 (9.2)	602 (12.3)	683 (18.5)	1,701 (12.9)	342 (12.3)	126 (11.8)
Congestive heart failure (5)	0 (0.0)	2 (0.05)	1 (0.02)	5 (0.1)	4 (0.03)	0 (0.0)	0 (0.0)
Pulmonary hypertension (4)	0 (0.0)	1 (0.02)	2 (0.04)	3 (0.1)	4 (0.03)	1 (0.04)	2 (0.2)
Ischemic heart disease/ cardiac arrhythmia (3)	0 (0.0)	9 (0.2)	11 (0.2)	9 (0.2)	51 (0.4)	7 (0.3)	2 (0.2)
Congenital heart and/or valvular disease (4)	0 (0.0)	6 (0.1)	20 (0.4)	9 (0.2)	46 (0.3)	10 (0.4)	2 (0.2)
Placental abruption (4)	1 (0.5)	28 (0.6)	27 (0.6)	21 (0.6)	70 (0.5)	9 (0.3)	8 (0.8)
Autoimmune disease/lupus (2)	2 (1.0)	50 (1.1)	70 (1.4)	40 (1.1)	292 (2.2)	50 (1.8)	17 (1.6)
HIV/AIDS (2)	0 (0.0)	1 (0.02)	3 (0.06)	9 (0.2)	3 (0.02)	3 (0.1)	0
Sickle cell disease/bleeding disorder/coagulopathy/anticoagulation (3)	2 (1.0)	40 (0.9)	96 (2.0)	152 (4.1)	324 (2.5)	57 (2.0)	36 (3.4)
Epilepsy/cerebrovascular accident/neuromuscular disorder (2)	0 (0.0)	19 (0.04)	42 (0.9)	37 (1.0)	96 (0.7)	22 (0.8)	5 (0.5)
Chronic renal disease (1)	0 (0.0)	0 (0.0)	2 (0.04)	5 (0.1)	4 (0.03)	1 (0.04)	0 (0.0)
Asthma (1)	12 (6.4)	156 (3.5)	417 (8.5)	458 (12.4)	742 (5.6)	176 (6.3)	69 (6.5)
Diabetes on insulin (1)	9 (4.8)	109 (2.5)	47 (1.0)	45 (1.2)	128 (1.0)	48 (1.7)	18 (1.7)

© 2023 Wetcher CS et al. *JAMA Netw Open.*

Maternal age, years							
> 44 (3)	0 (0.0)	11 (0.3)	9 (0.2)	6 (0.2)	67 (0.5)	10 (0.4)	12 (1.1)
40-44 (2)	3 (1.6)	12 (0.3)	88 (1.8)	84 (2.2)	441 (3.3)	73 (2.6)	36 (3.4)
35-39 (1)	22 (11.7)	778 (17.6)	502 (10.2)	502 (13.6)	2,307 (17.5)	410 (14.7)	203 (19.1)
Substance use disorder (2)	1 (0.5)	8 (0.2)	55 (1.1)	56 (1.5)	112 (0.8)	26 (0.9)	15 (1.4)
Alcohol abuse (1)	0 (0.0)	6 (0.1)	14 (0.3)	16 (0.4)	39 (0.3)	9 (0.3)	6 (0.6)
Body mass index (BMI), kg/m^2							
>50 (3)	1 (0.5)	30 (0.7)	60 (1.2)	69 (1.9)	135 (1.0)	27 (1.0)	10 (0.9)
40-49.9 (2)	7 (3.7)	109 (2.5)	332 (6.8)	438 (11.9)	704 (5.3)	151 (5.4)	68 (6.4)

Data are presented as n (%)

AIDS, acquired immunodeficiency syndrome; HIV, human immunodeficiency virus; OB-CMI, obstetric comorbidity index

[a] OB-CMI components excluded from study: multiple gestation, intrauterine fetal demise, placenta previa/suspected accreta, previous cesarean delivery/myomectomy

© 2023 Wetcher CS et al. *JAMA Netw Open.*

eTable 3. Cesarean delivery indications stratified by obstetrical comorbidity index (OB-CMI) score group and race and ethnicity group

Characteristic	All Patients[a]	American Indian or Alaskan Native	Asian or Pacific Islander	Hispanic	Non-Hispanic Black	Non-Hispanic White	Other or Multiracial	Declined or Unknown
All cesareans	n=8,632	n=50	n=1,194	n=1,371	n=1,273	n=3,566	n=848	n=330
Abnormal fetal status	4,328 (50.2)	33 (66.0)	669 (56.1)	662 (48.3)	778 (61.2)	1,582 (44.4)	436 (51.4)	168 (50.9)
Elective (maternal request)	909 (10.5)	4 (8.0)	92 (7.7)	144 (10.5)	74 (9.5)	470 (13.2)	92 (10.8)	33 (10.0)
Maternal condition	272 (3.2)	1 (2.0)	23 (1.9)	45 (3.3)	42 (3.3)	133 (3.7)	18 (2.1)	10 (3.0)
Obstetrical	3,123 (36.2)	12 (24.0)	410 (34.3)	520 (37.9)	379 (29.8)	1,381 (38.7)	302 (35.6)	119 (36.1)
OB-CMI = 0 cesareans	n=3,762	n=22	n=595	n=647	n=450	n=1,516	n=389	n=143
Abnormal fetal status	2,018 (53.7)	14 (63.6)	349 (58.7)	330 (51.1)	300 (66.7)	730 (48.2)	219 (56.3)	76 (53.1)
Elective (maternal request)	352 (9.4)	3 (13.6)	39 (6.6)	73 (11.3)	25 (5.6)	164 (10.8)	37 (9.5)	11 (7.7)
Maternal condition	89 (2.4)	0 (0.0)	9 (1.5)	18 (2.8)	10 (2.2)	43 (2.8)	4 (10.3)	5 (3.5)
Obstetrical	1,303 (34.6)	5 (22.7)	198 (33.3)	226 (34.9)	115 (25.6)	579 (38.2)	129 (33.2)	51 (35.7)
OB-CMI = 1 cesareans	n=1,368	n=9	n=244	n=170	n=181	n=572	n=138	n=54
Abnormal fetal status	692 (50.6)	7 (77.8)	132 (54.1)	98 (57.6)	112 (62.2)	250 (43.7)	62 (44.9)	31 (57.4)
Elective (maternal request)	178 (13.0)	1 (11.1)	25 (10.2)	20 (11.8)	8 (4.4)	95 (16.6)	20 (14.5)	9 (16.7)
Maternal condition	38 (2.8)	0 (0.0)	4 (1.6)	3 (1.8)	4 (2.2)	24 (4.2)	2 (1.4)	1 (1.9)
Obstetrical	460 (33.6)	1 (11.1)	82 (33.6)	49 (28.8)	57 (31.5)	203 (35.5)	54 (39.1)	13 (24.1)
OB-CMI = 2 cesareans	n=1,356	n=13	n=156	n=204	n=228	n=575	n=137	n=43
Abnormal fetal status	651 (48.0)	8 (61.5)	91 (58.3)	96 (47.1)	130 (57.3)	236 (41.0)	69 (50.4)	21 (48.8)
Elective (maternal request)	171 (12.6)	0 (0.0)	11 (7.1)	17 (8.3)	18 (7.9)	100 (17.4)	18 (13.1)	7 (16.3)
Maternal condition	33 (2.4)	0 (0.0)	3 (1.9)	7 (3.4)	7 (3.1)	13 (2.3)	3 (2.2)	0 (0.0)
Obstetrical	501 (36.9)	5 (38.5)	51 (32.7)	84 (41.2)	73 (32.0)	226 (39.3)	47 (34.3)	15 (34.9)

© 2023 Wetcher CS et al. *JAMA Netw Open.*

OB-CMI = 3 cesareans	n=644	n=1	n=73	n=82	n=94	n=299	n=65	n=30
Abnormal fetal status	322 (50.1)	0 (0.0)	35 (47.9)	36 (43.9)	63 (67.0)	145 (48.7)	31 (47.7)	12 (40.0)
Elective (maternal request)	72 (11.2)	0 (0.0)	11 (15.1)	10 (12.2)	5 (5.3)	37 (12.4)	6 (9.2)	3 (10.0)
Maternal condition	20 (3.1)	0 (0.0)	1 (1.4)	1 (1.2)	2 (2.1)	12 (4.0)	3 (4.6)	1 (3.3)
Obstetrical	230 (35.7)	1 (100.0)	26 (35.6)	35 (42.7)	24 (25.5)	105 (35.1)	25 (38.5)	14 (46.7)
OB-CMI ≥ 4 cesareans	n=1,502	n=5	n=126	n=268	n=320	n=604	n=119	n=60
Abnormal fetal status	645 (43.0)	4 (80.0)	62 (49.2)	102 (38.1)	173 (54.1)	221 (36.6)	55 (46.2)	28 (46.7)
Elective (maternal request)	136 (9.1)	0 (0.0)	6 (4.8)	24 (9.0)	18 (5.6)	74 (12.3)	11 (9.2)	3 (5.0)
Maternal condition	92 (6.1)	1 (20.0)	6 (4.8)	16 (6.0)	19 (5.9)	41 (6.8)	6 (5.0)	3 (5.0)
Obstetrical	629 (41.9)	0 (0.0)	52 (41.3)	126 (47.0)	110 (34.4)	268 (44.4)	47 (39.5)	26 (43.3)

Data are presented as n (%)

© 2023 Wetcher CS et al. *JAMA Netw Open.*

eTable 4. Cesarean delivery for abnormal fetal status stratified by obstetrical comorbidity index (OB-CMI) score group and race and ethnicity group

Characteristic	All Patients[a]	Asian or Pacific Islander	Hispanic	Non-Hispanic Black	Non-Hispanic White	Other or Multiracial	P value[b]
All cesareans							
Abnormal fetal status	4,328 (50.2)	669 (56.1)	662 (48.3)	778 (61.2)	1,582 (44.4)	436 (51.4)	<0.001
Other indication	4,297 (49.8)	524 (43.9)	708 (51.7)	493 (38.8)	1,982 (55.6)	412 (48.6)	
OB-CMI = 0							
Abnormal fetal status	2,018 (53.7)	349 (58.8)	330 (51.1)	300 (66.7)	730 (48.2)	219 (56.3)	<0.001
Other indication	1741 (46.3)	245 (41.2)	316 (48.9)	150 (33.3)	785 (51.8)	170 (43.7)	
OB-CMI = 1							
Abnormal fetal status	692 (50.6)	132 (54.1)	98 (57.6)	112 (62.2)	250 (43.7)	62 (44.9)	<0.001
Other indication	675 (49.4)	112 (45.9)	72 (42.4)	68 (37.8)	322 (56.3)	76 (55.1)	
OB-CMI = 2							
Abnormal fetal status	651 (48.0)	91 (58.3)	96 (47.1)	130 (57.3)	236 (41.0)	69 (50.4)	<0.001
Other indication	704 (52.0)	65 (41.7)	108 (52.9)	97 (42.7)	339 (59.0)	68 (49.6)	
OB-CMI = 3							
Abnormal fetal status	322 (50.1)	35 (47.9)	36 (43.9)	63 (67.0)	145 (48.7)	31 (47.7)	0.01
Other indication	321 (49.9)	38 (52.1)	46 (56.1)	31 (33.0)	153 (51.3)	34 (52.3)	
OB-CMI ≥ 4							
Abnormal fetal status	645 (43.0)	62 (49.2)	102 (38.1)	173 (54.1)	221 (36.6)	55 (46.2)	<0.001
Other indication	856 (57.0)	64 (50.8)	166 (61.9)	147 (45.9)	383 (63.4)	64 (53.8)	
**P value[c]**	<0.001	0.15	<0.001	0.01	<0.001	0.10	

Data are presented as n (%)
[a] Includes American Indian or Alaskan Native (n=50 cesareans) and declined/unknown race and ethnicity (n=329 cesareans); these groups are not shown in stratified results.
[b] Chi square analysis used to examine indications across race and ethnicity groups within a given OB-CMI score group
[c] Chi square analysis used to examine indications across OB-CMI score groups within a given race and ethnicity group

© 2023 Wetcher CS et al. *JAMA Netw Open.*

eTable 5. Perinatal outcomes by obstetrical comorbidity index (OB-CMI) score group

	OB-CMI Score Group					
	**0**	**1**	**2**	**3**	**≥ 4**	
**Characteristic**	(n=17,055)	(n=4,276)	(n=3,971)	(n=1,759)	(n=3,192)	**P value**
Birthweight, grams	3,308 ± 426	3,316 ± 438	3,302 ± 458	3,290 ± 455	3,233 ± 485	<0.001
Low birth weight[a]	427 (2.5)	106 (2.5)	154 (3.9)	69 (3.9)	195 (6.1)	<0.001
Small for gestational age[b]	2,053 (12.0)	523 (12.2)	480 (12.1)	225 (12.8)	485 (15.2)	<0.001
5-minute APGAR <7	145 (0.9)	26 (0.6)	48 (1.2)	24 (1.4)	67 (2.1)	<0.001
Postpartum hemorrhage with blood transfusion	385 (2.3)	95 (2.2)	115 (2.9)	67 (3.8)	210 (6.6)	<0.001

Data are presented as n (%) and mean ± standard deviation.
[a] Less than 2,500 grams
[b] Less than 10th percentile for gestational age at birth

© 2023 Wetcher CS et al. *JAMA Netw Open.*
