# Supplementary material for: Association of Maternal Comorbidity Burden With Cesarean Birth Rate Among Nulliparous, Term, Singleton, Vertex Pregnancies
Source: JAMA Netw Open. 2023 Oct 19;6(10):e2338604. doi: 10.1001/jamanetworkopen.2023.38604 (PMC10587795; doi:10.1001/jamanetworkopen.2023.38604)
Supplement: Supplement 2. — Data Sharing Statement [file jamanetwopen-e2338604-s002.pdf]

Data Sharing Statement

Wetcher. Association of Maternal Comorbidity Burden With Cesarean Birth Rate Among Nulliparous, Term, Singleton, Vertex Pregnancies. *JAMA Netw Open*. Published October 19, 2023. doi:10.1001/jamanetworkopen.2023.38604

Data
Data available: No
